# A Rare Case of Idiopathic Rapid Gastric Emptying

**DOI:** 10.7759/cureus.106710

**Published:** 2026-04-09

**Authors:** Saumyaa Vohra, Yohann E Pinto, Kaiser Raja

**Affiliations:** 1 Internal Medicine, King's College Hospital London - Dubai, Dubai, ARE; 2 Gastroenterology and Hepatology, King's College Hospital London - Dubai, Dubai, ARE

**Keywords:** dumping-like syndrome, gastrointestinal motility disorder, idiopathic rapid gastric emptying, ocreotide, reactive hypoglycemia

## Abstract

Idiopathic rapid gastric emptying (IRGE), also called idiopathic dumping syndrome, is a rare gastrointestinal (GI) motility disorder marked by an abnormally accelerated gastric emptying with no history of gastric surgery, diabetes mellitus, or other structural anomalies of the stomach or small intestine. This disorder has a similar presentation to classic dumping syndrome. Yet, it happens in patients with an intact stomach and pylorus, with no evidence of previous gastric surgeries. Due to its similarity with other endocrine hypoglycemic syndromes and functional GI disorders, IRGE remains misdiagnosed and/or underreported. Thus, it is a significant diagnostic challenge for physicians.

We present a case of a 21-year-old female with a chronic history of postprandial diarrhea, carbohydrate intolerance, and symptomatic postprandial hypoglycemia. She had no history of prior gastric surgery, and her investigations, including endoscopy and radiology, demonstrated normal findings. Gastric emptying scintigraphy (GES) revealed 100% gastric emptying at 60 minutes (retained meal value of less than 70% at 30 minutes or less than 30% at 1 hour is indicative of rapid gastric emptying). Based on these findings and after exclusion of other differential diagnoses, a diagnosis of IRGE was established.

This case highlights the importance of identifying IRGE as a distinct clinical entity that causes postprandial hypoglycemia and GI symptoms. Early recognition can help in timely diagnosis and targeted therapy.

## Introduction

The clinical manifestations of rapid gastric emptying (RGE) are usually non-specific, and the underlying pathophysiology in patients without previous gastric surgery remains unknown. It is typically diagnosed using gastric emptying scintigraphy (GES). The symptoms of idiopathic rapid gastric emptying (IRGE) are often vague and can oddly resemble those of delayed gastric emptying [1]. Clinicians should suspect RGE when patients present with a distinct postprandial symptom pattern. This includes early dumping symptoms, including bloating, nausea, and diarrhea, which can occur with vasomotor or hypoglycemic symptoms like tremor, sweating, lethargy, and confusion. These symptoms usually resolve after ingestion of any sweet food or drink [2-4]. Importantly, a history of gastric surgery or other structural anomalies of the stomach or small intestine should be ruled out before establishing a diagnosis of IRGE.

IRGE is difficult to diagnose since its clinical presentation closely resembles several other conditions, including endocrine hypoglycemic syndromes or other functional gastrointestinal disorders (FGIDs). As a result, patients may undergo extensive investigations before they reach a final diagnosis.

In contrast to post-surgical RGE, IRGE is understudied, and there is limited literature on the clinical presentation, pathophysiology, and treatment options. Xiao et al. found that 8% of patients referred for GES had IRGE in a single-center observational study [2]. Therefore, IRGE remains an uncommon and likely underrecognized disorder, with the published literature primarily consisting of case reports, small case series and limited observational data. Most cases describe young to middle-aged Caucasian adult patients presenting with chronic postprandial GI and autonomic symptoms.

We present a case of IRGE in a 21-year-old female who presented with a chronic history of postprandial diarrhea, carbohydrate intolerance, and symptomatic hypoglycemia.

## Case presentation

A 21-year-old Caucasian woman presented with a five-year history of recurrent postprandial abdominal cramps, diarrhea, nausea and lightheadedness. On detailed questioning with the patient's mother, it was noted that the patient had experienced similar but milder intermittent symptoms since the age of four years, although these were infrequent and did not need medical evaluation at the time. The symptoms became more frequent and clinically significant during late adolescence, progressing over the next five years.

The patient reported recurrent post-meal episodes of watery diarrhea (3-5 times/day), often occurring within 30-60 minutes of eating carbohydrate-rich meals. These episodes were accompanied by abdominal cramping, early satiety, nausea, and occasional vomiting. She also described lightheadedness, tremors, palpitations, and fatigue 40 to 60 minutes after meals. Her symptoms were often relieved with the intake of sweet fruit juices or glucose tablets. She denied any symptoms during the fasting state or at night. In the one-year prior to presentation, she had lost about 5 kg of weight. She was documented to have repeated postprandial glucose readings of 2.3-2.8 mmol/L (normal fasting glucose 3.9-5.5 mmol/L) measured at home with a glucometer. 

She had a past medical history of a pituitary microadenoma for which she had a transsphenoidal surgery at the age of 16 years, following which she was in clinical and biochemical remission. She also had hypothyroidism managed with levothyroxine. There were no menstrual irregularities. There was no family history of endocrine tumors, hematologic, or gastrointestinal diseases. Physical examination was unremarkable, including a normal BMI of 20 kg/m^2^.

Laboratory investigations revealed normal blood counts and serum chemistries. Iron studies, vitamin D and B12 levels were normal. Celiac serology was negative. Glycemic evaluation showed HbA1c of 4.3% and fasting glucose of 4.3 mmol/L. However, C-peptide was elevated at 2.0 nmol/L (0.2-0.9) with an insulin level of 20.1 mIU/L (2-25). Insulin receptor antibodies were negative. Additional endocrine investigations demonstrated normal morning cortisol at 365 nmol/L (200-700 nmol/L), adrenocorticotropic hormone (ACTH) at 18 pg/mL (7-63 pg/mL), and thyroid-stimulating hormone (TSH) at 1.3 µIU/mL (0.79-4.68 µIU/mL) (Table 1).

**Table 1:** Laboratory findings at presentation. ACTH: Adrenocorticotropic hormone; TSH: Thyroid-stimulating hormone

Test	Result	Reference range
Hemoglobin	15.0 g/dl	115-15.5 g/dl
Mean Corpuscular Volume (MCV)	92 fL	80-96 fL
White blood cell count	7.8*10^9^/L	4-11*10^9^/L
Platelet count	276 *10^9^/L	150-450*10^9^/L
Ferritin	130 ng/ml	15-150 ng/ml
HbA1c	4.3%	<5.7%
Fasting glucose	4.3 mmol/L	3.8-5.6 mmol/L
C-peptide	2.0 nmol/L	0.2-0.9 nmol/L
Insulin	20.1 mIU/L	2-25 mIU/L
Insulin receptor antibodies	Negative	
Cortisol (AM)	365 nmol/L	200-700 nmol/L
ACTH	18 pg/mL	7-63 pg/mL
TSH	1.3 µIU/mL	0.79-4.68 µIU/mL
Vitamin D	112 nmol/L	75-250 nmol/L
Celiac serology	Negative	-

**Figure 1:**
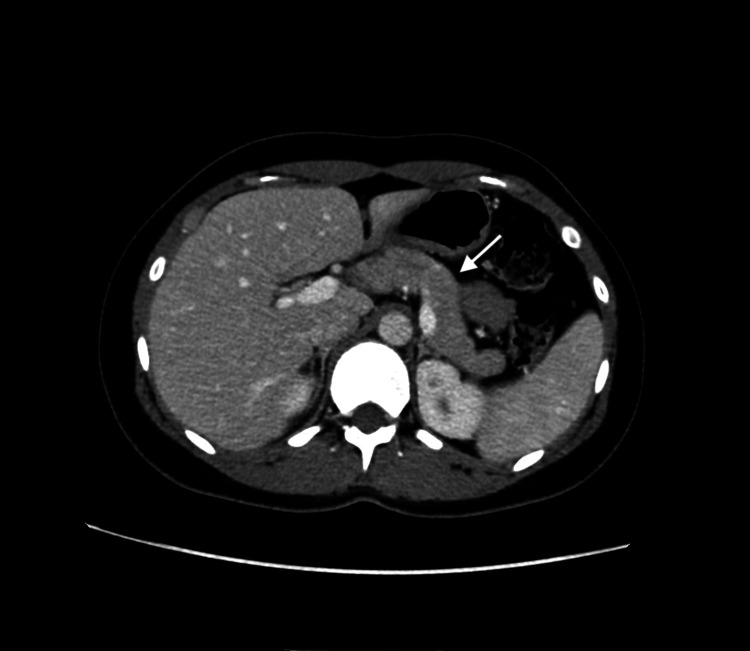
Contrast-enhanced axial CT of the abdomen displaying normal findings of the pancreas. Axial contrast-enhanced CT of the abdomen demonstrating normal pancreatic morphology without focal lesions, helping exclude insulinoma.

**Figure 2:**
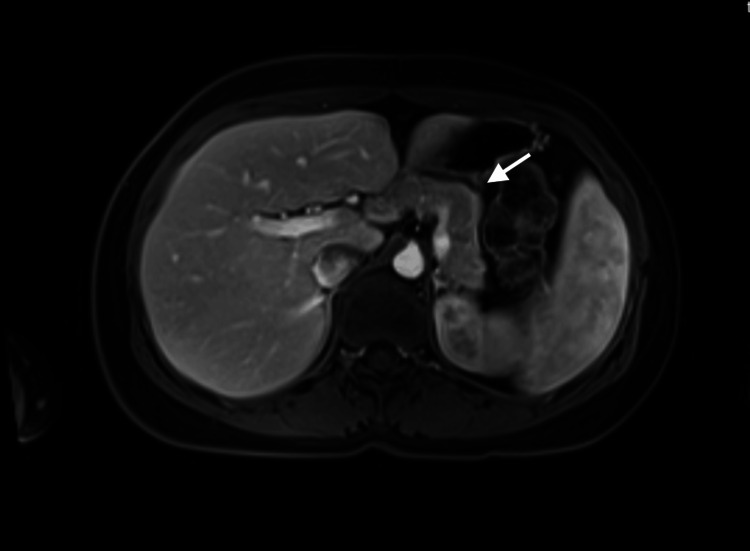
Contrast-enhanced axial MRI of the abdomen displaying normal findings of the pancreas. Axial contrast-enhanced MRI of the abdomen demonstrating normal pancreatic morphology without focal lesions, helping exclude insulinoma.

Radiological and endoscopic investigations were performed to exclude structural and neoplastic causes. Computed tomography (CT) (Figure 1) and magnetic resonance imaging (MRI) (Figure 2) of the abdomen with contrast were unremarkable, effectively ruling out insulinoma. 

Upper and lower GI endoscopy showed normal mucosa with no structural abnormalities. A GES was then performed using the standardized solid-meal protocol. The patient received a radiolabeled meal consisting of 1 mCi technetium-99m sulfur colloid mixed with scrambled eggs. After ingesting the meal, a combination of dynamic and static anterior and posterior abdominal images was obtained for computer- assisted quantitation over a period of 1.5 hours. The GES scan demonstrated 100% gastric emptying at 60 minutes. According to established scintigraphic criteria, RGE is defined as <70% gastric retention at 30 minutes or <30% retention at 1 hour. These findings therefore confirmed markedly accelerated gastric emptying.

The patient’s clinical history, along with investigations like endoscopy and radiology, ruled out other potential differential diagnoses, including post-surgical dumping syndrome, autoimmune insulin syndrome, adrenal insufficiency, thyroid disorders, diabetes mellitus, nesidioblastosis and insulinoma. Therefore, the final diagnosis was IRGE, causing reactive hypoglycemia and postprandial diarrhea.

This patient was initially managed with dietary modification, including small, frequent meals, low glycemic index carbohydrates and avoidance of liquids during meals. In addition, acarbose was started to delay carbohydrate absorption. After six months on this treatment, the patient reported approximately 50% reduction in postprandial diarrhea, but symptoms of early satiety, nausea, and postprandial hypoglycemia persisted. Due to persistent symptoms, treatment was escalated to subcutaneous octreotide 100 mcg three times daily, 45 minutes before major meals. Within a week of starting this treatment, the patient demonstrated a marked reduction in diarrhea and an approximate 50% reduction in hypoglycemic episodes. Objective follow-up revealed a 3 kg weight gain over three months and improvement in bowel frequency to 1-2 stools per day. Over six months of follow-up on octreotide therapy, the patient was clinically stable with complete resolution of symptomatic hypoglycemia. Home glucose monitoring demonstrated postprandial glucose levels ranging between 3.6-5.4 mmol/L (normal fasting glucose 3.9-5.5 mmol/L), and the patient maintained stable nutritional status without further weight loss.

## Discussion

IRGE is an underrecognized condition due to its rarity and similarity with other GI and endocrine origin disorders. When there is no history of previous gastric surgery or other structural abnormality, physicians do not usually consider accelerated gastric emptying as the initial differential, resulting in the patient undergoing extensive investigations to reach a diagnosis. In addition to this, many times, it is misdiagnosed as a hypoglycemic syndrome of endocrine origin, which may cause further diagnostic delay.

Symptoms of the IRGE

Symptoms are classified into early and late dumping. Early dumping happens within one hour of eating and is associated with hypotension and can occasionally cause syncope. Tachycardia, palpitations, flushing, and sweating are signs of an autonomic stress reaction that ensues. Abdominal distention, severe cramps, borborygmi, nausea, and diarrhea are among the GI symptoms that may happen in early dumping. Late dumping is characterized by reactive hypoglycemia, which starts one to three hours after a meal. Reactive hypoglycemia is suggested by signs including palpitations, tremor, sweating, and irritability. When it is associated with confusion and presyncope, it is considered neuroglycopenia [3]. A glucose level of ≤3.9 mmol/L or less than fasting glucose following an oral glucose load, or a plasma glucose level of less than 3 mmol/L during the postprandial phase, is used to confirm this [4]. Patients with dumping commonly have only early dumping symptoms, but some can also have a combination of early and late dumping symptoms. However, it is uncommon for a patient to experience only symptoms of late dumping [3]. Our patient experienced a combination of both early and late dumping symptoms.

Differential diagnosis and misdiagnosis

Since IRGE is a diagnosis of exclusion, many diseases must be ruled out before establishing a final diagnosis of IRGE [1,4]. When a patient presents with GI symptoms and postprandial hypoglycemia, a number of other diagnoses need to be considered. These include FGIDs such as irritable bowel syndrome, insulinoma, autoimmune insulin syndrome and nesidioblastosis. In this instance, laboratory tests were conducted to assess potential endogenous hyperinsulinism. These samples were not taken during reported hypoglycemia, despite the patient's increased C-peptide levels. Therefore, hyperinsulinism cannot be conclusively confirmed by this data, even though it may indicate higher endogenous insulin secretion. Testing for insulin autoantibodies was negative, making autoimmune insulin syndrome unlikely. Since there was no pancreatic lesion seen on contrast-enhanced MRI imaging, insulinoma seems unlikely. An upper GI endoscopy revealed normal mucosa and no structural anomalies that would explain the patient's symptoms. Additional endocrine evaluation, including thyroid function testing and cortisol levels, was within normal limits, helping exclude alternative metabolic causes of hypoglycemia.

Insulinoma is a rare functional neuroendocrine tumor that develops from the pancreatic islet cells. Fasting hypoglycemia, palpitations, tremors, and neuroglycopenic symptoms such as confusion and seizures are among the symptoms. A plasma glucose concentration of less than 55 mg/dL, an insulin level of at least 3 µU/mL, and a C-peptide level of at least 0.6 ng/mL are diagnostic criteria. About 85% of insulinomas are detected by MRI, and after gadolinium is administered, they often enhance homogenously [5]. The pathophysiology of nesidioblastosis-induced hypoglycemia in adults is unknown. Increased periductular islets, larger β-cell nuclei, and increased islet size and quantity were among the early histopathologic findings of nesidioblastosis [6]. Autoimmune insulin syndrome is also a rare cause of hypoglycemic episodes characterized by increased levels of insulin autoantibodies [7]. Post-bariatric dumping syndrome’s clinical presentation is very similar to IRGE but only occurs in patients who have surgically altered gastric anatomy. Other secondary causes should also be ruled out before concluding the diagnosis of IRGE, including diabetic autonomic neuropathy, hyperthyroidism, functional dyspepsia, and medication-induced acceleration of gastric motility. This patient experienced no signs of fasting hypoglycemia, has never undergone abdominal surgery, had normal imaging scans of the abdomen, and had a negative antibody screen. Therefore, other diagnoses with similar presentation were excluded.

Diagnostic confirmation with GES

More recently, GES is being used to assess for RGE. A retained meal value of less than 70% at 30 minutes or less than 30% at 1 hour indicates RGE [8]. The patient in our case showed 100% emptying at 60 minutes, which is abnormal and lines up with the diagnosis of RGE. If GES is included as one of the initial tests for evaluating postprandial hypoglycemia, it can decrease further unnecessary endocrine tests as well as expedite diagnosis.

Pathophysiology of IRGE

Research has not been done on the mechanisms of RGE in individuals who have an "idiopathic" disorder. Patients with “idiopathic” RGE have a motor disturbance characterized by increased postprandial gastric motility but normal fasting volumes and postprandial accommodation. RGE may result from either increased "propulsive" forces (such as gastric contractility) or decreased "resistive" forces (such as decreased pyloric tone and non-propulsive duodenal contractions), as gastric emptying is typically controlled by a balance between propulsive and resistive forces. Furthermore, about 40% of patients with functional dyspepsia have reduced gastric accommodation, which may predispose them to increased gastric pressures, which accelerate gastric emptying. Thus, it is postulated that greater gastric motility and decreased postprandial accommodation are linked to IRGE [9]. The fast delivery of food to the small intestine produces rapid glucose absorption, which results in hyperglycemia and increased GLP-1 release. This phenomenon is known as the "late dumping syndrome," and it happens one to three hours after meals. Reactive hypoglycemia is caused by excessive insulin release, which is explained by hyperglycemia and elevated GLP-1 secretion [10]. Although this patient had elevated C-peptide levels, endogenous hyperinsulinism cannot be confirmed because these measures were not taken during documented hypoglycemia. However, the finding aligns with the incretin-mediated physiology described in dumping-like syndromes. In addition, the diarrhea and vasomotor symptoms that set in postprandially may be due to the osmotic shift within the small bowel.

Management of IRGE

Smaller, more frequent meals, roughly six times a day, are advised. Fluid intake should be postponed for at least half an hour. Alcohol and rapidly absorbable carbohydrates should be avoided, however eating foods high in protein and fiber is advised. The third step on the therapeutic ladder is pharmacological interventions. However, there is currently no recognized treatment for dumping syndrome. Currently, there are two options, which include acarbose and somatostatin analogues for severe cases.

Alpha-glucosidase inhibitors like acarbose reduce intraluminal digestion of carbohydrates within the duodenum. As a result, it treats postprandial hypoglycemia in late dumping syndrome [11]. However, our patient did not show sufficient improvement with acarbose.

Somatostatin analogs like octreotide provide therapeutic benefit for dumping syndrome through several mechanisms. It does this by delaying gastric emptying, slowing small bowel transit, suppressing GI hormone release, inhibiting insulin secretion, and decreasing postprandial vasodilation. Octreotide can be used either subcutaneously three times a day or intramuscularly every two to four weeks [11]. In another reported case about RGE, dietary modifications and acarbose improved symptoms of late dumping syndrome. However, diet modification alone failed to treat the symptoms of early dumping syndrome. Therefore, further management with octreotide was implemented. Octreotide proved effective for the symptoms of early dumping syndrome in this case report, especially postprandial tachycardia [12]. It is important to note that follow-up of patients on long-term octreotide therapy requires routine monitoring of gallstone disease, fat-soluble vitamins due to deficiency, and glucose intolerance. This is in accordance with the long-term somatostatin analogue safety profiles [13]. Our patient’s symptoms were controlled effectively only once octreotide was added to the treatment regimen. It proved effective for symptoms of both early and late dumping syndrome.

Marked improvement in symptoms after octreotide therapy provides an indirect diagnostic clue, supporting a motility- and incretin-mediated mechanism rather than pancreatic β-cell pathology. This therapeutic confirmation has been described in idiopathic dumping-like syndromes and directs clinicians towards the diagnosis of IRGE.

Other reported therapy options include diazoxide, which is a potassium channel activator that affects hypoglycemia and is therefore used in late dumping syndrome [11]. Calcium channel blockers (CCBs) delay gastric emptying by inhibiting gastric smooth muscle contraction. Although results from research by Yavorski et al. revealed no significant difference in gastric emptying rates after pretreatment with verapamil or diltiazem when compared with no premedication [14]. The evidence about the effectiveness of diazoxide and CCBs for the management of IRGE is limited.

Outcome of IRGE and follow-up

There is limited data regarding the prognosis of IRGE since it is a rare condition. Available evidence about the disease reveals that most patients have symptoms that persist.

Childhood onset

IRGE presenting in childhood, as seen in this patient, is uncommon and widens our understanding of IRGE’s pathophysiology. Most of the reported cases of the disease include young and middle-aged adults with no mention of a childhood history of similar symptoms. Childhood onset of the disease may hint towards the possibility of congenital abnormalities of the enteric nervous system. Pediatric presentation of IRGE resembles other functional GI pathologies, which results in delayed diagnosis of the condition.

## Conclusions

IRGE is an uncommon clinical entity characterized by severe dysfunction of GI motility that may present with postprandial hypoglycemia, diarrhea, and autonomic symptoms. Patients with this disorder have no history of previous gastric surgery or structural disease. IRGE draws similarity with endocrine hypoglycemic syndromes as well as FGIDs, which leads to extensive investigations and delays the diagnosis of the condition. This case highlights the importance of considering accelerated gastric emptying as a differential in patients with a history of recurrent postprandial symptoms when more common endocrine and structural causes have been ruled out. GES remains an important investigation to diagnose the condition. Increased clinical awareness of IRGE can help in the earlier recognition and appropriate management of this potentially underrecognized disorder.
